# Chelerythrine Inhibits TGF-β-Induced Epithelial–Mesenchymal Transition in A549 Cells via RRM2

**DOI:** 10.3390/ph18071036

**Published:** 2025-07-12

**Authors:** Jinlong Liu, Mengran Xu, Liu Han, Yuxuan Rao, Haoming Han, Haoran Zheng, Jinying Wu, Xin Sun

**Affiliations:** School of Pharmaceutical Sciences, Jilin Medical University, No. 5, Jilin Street, Fengman District, Jilin 132013, China; jinlongliu@jlmu.edu.cn (J.L.); xmr95mail@163.com (M.X.); hanliu@jlmu.cn (L.H.); 15070451553@163.com (Y.R.); hhm3240711@163.com (H.H.); 13614374349@163.com (H.Z.)

**Keywords:** chelerythrine, non-small-cell lung cancer, epithelial–mesenchymal transition, RRM2

## Abstract

**Background:** The mechanisms underlying the metastasis of non-small-cell lung cancer (NSCLC) have long been a focal point of medical research. The anti-tumor effects of chelerythrine (CHE) have been confirmed; however, its ability to inhibit tumor metastasis and the underlying mechanisms remain unknown. The aim of this study was to investigate the inhibitory effects and molecular mechanisms of CHE on transforming growth factor-beta (TGF-β)-induced epithelial–mesenchymal transition (EMT). **Methods**: Wound healing and Transwell assays were employed to evaluate TGF-β-induced migration in A549 cells and the inhibitory effects of CHE. Ribonucleotide reductase subunit M2 (RRM2) expression levels were detected via Western blot and immunofluorescence staining. Western blot and RT-qPCR were used to examine the expression levels of EMT-related markers. Animal experiments were conducted to analyze the role of RRM2 in the CHE inhibition of TGF-β-induced lung cancer metastasis. **Results**: This study found that TGF-β treatment enhanced the metastasis of A549 cells, while CHE inhibited the expression of TGF-β-induced EMT-related transcription factors by RRM2, thereby suppressing tumor cell migration (*p* < 0.05). Furthermore, the oral administration of CHE inhibited the metastasis of A549 cells to the lungs from the tail vein in mice, consistent with in vitro findings. Despite the high doses of CHE used, there was no evidence of toxicity. **Conclusions**: Our data reveal the mechanism of the anti-metastatic effects of CHE on TGF-β-induced EMT and indicate that CHE can be used as an effective anti-tumor treatment.

## 1. Introduction

In recent years, cancer treatment has remained a challenge, with lung cancer emerging as the leading cause of cancer-related deaths worldwide [1,2]. Approximately 2 million new cases and 1.76 million deaths occur each year, with the highest incidence found in 20 countries, including the United States, Canada, and China [3,4]. Non-small-cell lung cancer (NSCLC) accounts for about 85% of all lung cancer cases and continues to be a significant cause of cancer-related deaths globally [5,6]. Even after surgery, chemotherapy, radiotherapy, and targeted therapies, the five-year survival rate remains disappointing. Compared to patients with carcinoma in situ, those with lung cancer that has metastasized to distant sites have an even lower survival rate [7,8,9]. Therefore, selecting effective treatment drugs, understanding the molecular mechanisms of lung cancer metastasis, and identifying potential therapeutic targets to reduce metastasis are crucial for improving the survival rates and quality of life of lung cancer patients.

The metastasis of tumor cells is associated with epithelial–mesenchymal transition (EMT), a process whereby epithelial cells acquire mesenchymal characteristics [10]. EMT is crucial for the early transformation of tumors into invasive malignancies [11]. Notably, EMT is activated by a group of classical inductive factors, including the Snail, Slug, ZEB1, and ZEB2 factors, which commonly regulate each other and cooperate functionally [12]. There is a range of methods that can induce EMT, with the transforming growth factor-beta (TGF-β) family being a particularly common inducer [13]. TGF-β accelerates the epithelial plasticity of cancer cells during the EMT process, which is a prerequisite for invasion and dissemination. In NSCLC, TGF-β promotes proliferation, migration, and EMT through independent pathways [14,15,16]. Therefore, controlling the EMT process, particularly the TGF-β-regulated steps, is key to ensuring the efficacy of NSCLC treatments.

Recent studies have confirmed that the expression of regulating ribonucleotide reductase subunit M2 (RRM2), a small subunit of the ribonucleotide reductase complex, promotes the progression of various cancers, including NSCLC, ovarian cancer, and bladder cancer, by inducing the accumulation of deoxyribonucleoside triphosphates to support rapid cell division [17,18,19,20,21]. There is increasing evidence that RRM2 may be a promising target for lung cancer treatment. For example, a study by Rahman et al. [19] demonstrated that the regulation of RRM2 induces apoptosis in lung cancer cells by modulating Bcl-2 expression. Additionally, low expression levels of RRM2 may be used to assess the response of lung cancer to cisplatin-based chemotherapy [22]. Recent research has indicated that berberine hydrochloride and lncOCMRL1 silencing affect tumor cell metastasis by regulating RRM2 to inhibit EMT [23,24]. Therefore, targeting RRM2 to inhibit EMT will be an important strategy for inhibiting tumor metastasis.

**Figure 1:**
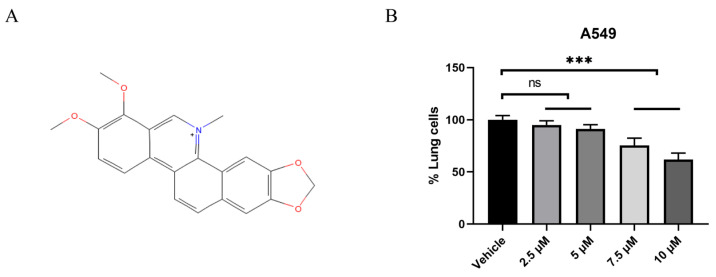
A549 cell proliferation after treatment with CHE. (**A**) The chemical structural formula of CHE. (**B**) A549 cells were treated with the specified concentration of CHE and cultured for 48 h, and cell viability was measured using a CCK8 assay. For multiple group comparisons, a one-way ANOVA was used. *** *p* < 0.001. ns: non-significance.

Chelerythrine (CHE) is a member of the *Chelidonium majus*. family, and it possesses a 2,3,7,8-tetrasubstituted benzophenanthridine structure (Figure 1). CHE is a naturally occurring alkaloid compound isolated from various plant sources, including Papaveraceae, Fumariaceae, and Rutaceae [25]. CHE exhibits various biological activities, such as antibacterial, anti-inflammatory, anti-parasitic, and anti-tumor effects [26,27,28,29,30,31,32,33,34]. CHE inhibits tumor cell proliferation through different pathways, such as MAPK, PI3K, and ErbB [29,35,36,37,38]. A recent study showed that CHE can reduce tumor volume and inhibit tumor EMT and that it has anti-cancer effects on colorectal and gastric cancers [39,40]. In NSCLC, CHE induced autophagy in A549 and NCI-H1299 cells and exhibited a good inhibitory effect on the characteristics of lung cancer tumor stem cells [23,41,42]. Current research on the CHE inhibition of NSCLC mainly focuses on proliferation aspects. However, the exact role of CHE in lung cancer metastasis remains unclear. Although some researchers have found that CHE chloride can inhibit NSCLC by downregulating β-catenin and inhibiting stem cell properties [42], the specific effects of CHE on NSCLC metastasis and EMT, and the mechanisms by which it influences these processes, remain unclear. Our study aims to fill this gap.

In this study, we evaluated the anti-metastatic effects of CHE on TGF-β-induced EMT and identified the molecular mechanisms underlying these effects. We found that CHE inhibits TGF-β-induced EMT and suppresses NSCLC migration and metastasis in vitro and in vivo, and we also found that RRM2 is involved in this process. The findings of this study introduce innovative therapeutic strategies and drugs for the clinical treatment of NSCLC.

## 2. Results

### 2.1. CHE Inhibited the Viability of A549 Cells

The chemical structural formula of CHE is shown in Figure 1A (https://pubchem.ncbi.nlm.nih.gov/#query=Chelerythrine, accessed on 3 June 2022). To investigate the role of CHE in A549 cells, we initially performed a CCK8 assay to evaluate its effects on cell viability. Our objective was to determine the appropriate concentration for subsequent experiments. Compared to the control group, 7.5 μM and 10 μM of CHE significantly reduced the survival rate of A549 cells, with this inhibitory effect being dose-dependent (*p* < 0.001; Figure 1B). Concentrations below 5 μM had a minimal impact on the viability of A549 cells (ns; Figure 1B). Consequently, we selected a concentration of ≤5 μM for further experimental investigation to assess the influence of CHE on the metastatic characteristics of A549 cells without inducing cytotoxicity.

### 2.2. CHE Inhibits TGF-β-Induced EMT

**Figure 2:**
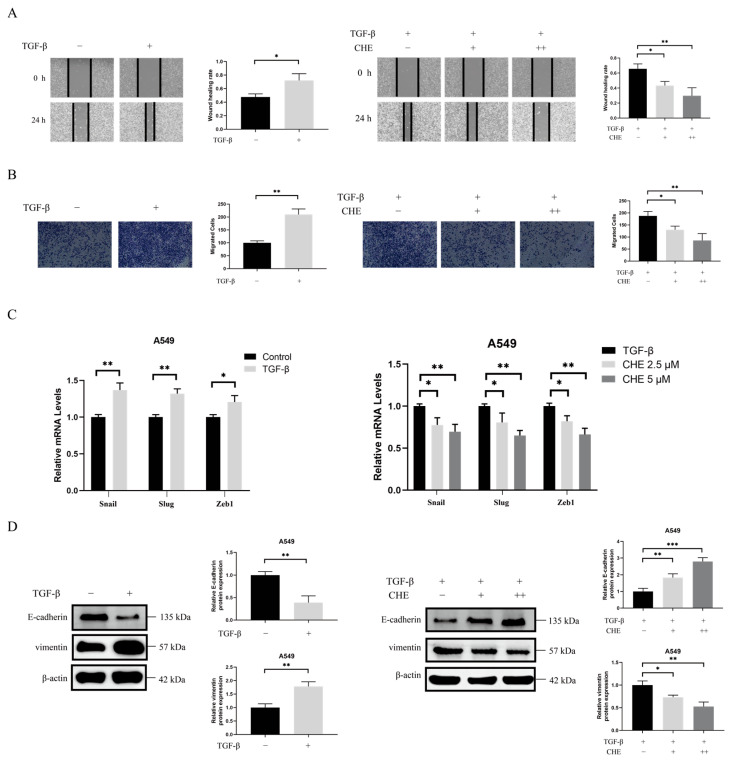
Effects of CHE on TGF-β-induced A549 cell migration and EMT. (**A**) Wound healing and (**B**) Transwell assays were used to assess tumor cell migration in A549 cells treated with TGF-β, with or without CHE. (**C**) A549 cells treated with TGF-β, with or without CHE, and changes in Snail, Slug, and ZEB1 mRNA levels. (**D**) A549 cells treated with TGF-β, with or without CHE, and E-cadherin and vimentin expression. Error bar, SD of three independent experiments. Comparison between two groups was performed using an unpaired Student’s *t*-test. For multiple group comparisons, a one-way ANOVA was used. * *p* < 0.05, ** *p* < 0.01, *** *p* < 0.001.

Research has indicated that EMT is a key step in the early stages of cancer metastasis and that TGF-β can induce EMT in cancer cells [43,44]. To evaluate the direct effect of TGF-β on EMT in A549 cells, we first assessed the impact of TGF-β on cell migration using wound healing and Transwell experiments. As shown in Figure 2A,B, TGF-β treatment significantly enhanced the migration of A549 cells. We then examined the levels of EMT biomarkers in A549 cells following TGF-β treatment. After stimulation with TGF-β, the expression of EMT-related genes (Snail, Slug, and ZEB1) increased in the A549 cells (*p* < 0.05; Figure 2C). TGF-β stimulation significantly decreased E-cadherin expression while increasing vimentin expression (*p* < 0.05; Figure 2D). Overall, these results support the notion that TGF-β enhances migration in lung tumors and directly promotes EMT.

We evaluated the inhibitory effect of CHE on TGF-β-induced A549 cell migration through wound healing and Transwell assays. As shown in Figure 2A,B, the results indicated that CHE treatment significantly inhibited the migration of TGF-β-induced A549 cells. Next, we examined the effects of CHE on EMT in A549 cells. CHE suppressed the increase in the expression of Slug, Snail, and ZEB1 induced by TGF-β (*p* < 0.05; Figure 2C). As illustrated in Figure 2D, CHE reversed the decrease in E-cadherin expression and the increase in vimentin expression induced by TGF-β.

### 2.3. A Therapeutic Target of LUAD Cells, RRM2, Was Suppressed by CHE

**Figure 3:**
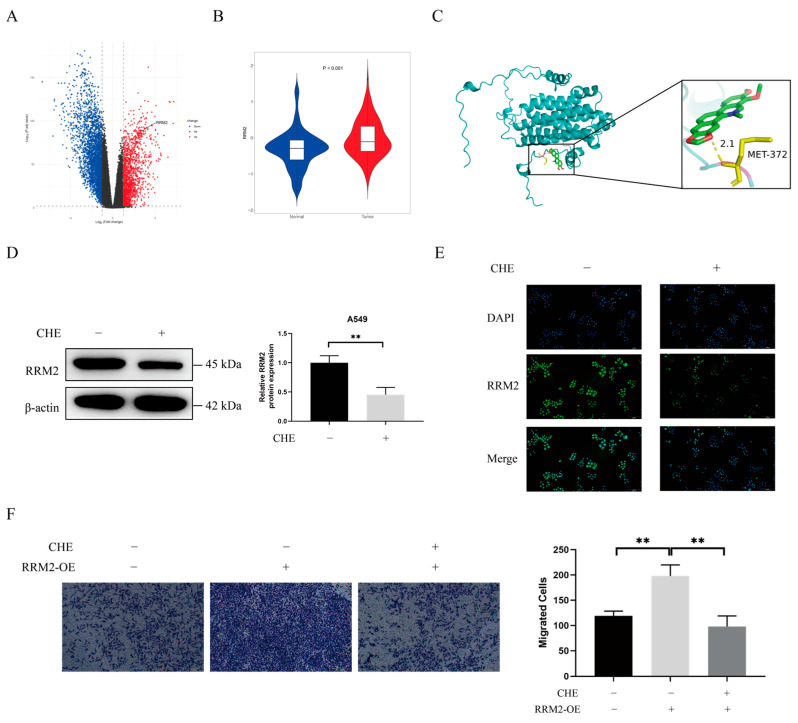
Effect of CHE on RRM2 in A549 cells. (**A**) A volcano plot showing that RRM2 was one of the upregulated miRNAs (PCAS). (**B**) RRM2 expression analysis using the PCAS database in LUAD (blue, normal group; red, tumor group). (**C**) Molecular docking model of CHE-RRM2 interaction (green: CHE; blue: RRM2). (**D**) RRM2 expression was analyzed using Western blot after A549 cells were treated with CHE. (**E**) After treatment with CHE, A549 cell morphology was examined, and the cells were fixed, permeabilized, and stained with anti-RRM2 antibody (green) and DAPI (blue). (**F**) A Transwell assay was used to assess tumor cell migration in A549 cells treated with or without RRM2-OE and CHE. All scale bars represent 200 µm. Error bar, SD of three independent experiments. Comparison between two groups was performed using an unpaired Student’s *t*-test. For multiple group comparisons, a one-way ANOVA was used. ** *p* < 0.01.

We queried bioinformatics databases to evaluate RRM2 expression in 16 human cancers. As shown in Figure S1A, we discovered that RRM2 expression did not significantly change in BRCA, CCRCC, or OV compared to normal tissues; however, it significantly increased in the remaining 13 cancer types. Interestingly, we observed that RRM2 was significantly upregulated in lung tumor tissues compared to in normal tissues (Figure 3A,B). In the overall survival analysis, a high RRM2 expression in LUAD predicted poor outcomes (Figure S1B). These data suggest that RRM2 is a valuable molecular biomarker for predicting prognosis and treatment efficacy in pan-cancer, particularly lung cancer.

To further understand how CHE inhibits RRM2 expression, we performed molecular docking experiments between CHE and RRM2. We used AutoDock-1.5.7 software to calculate the binding affinity between CHE and RRM2. The results showed that CHE binds tightly to RRM2 through hydrogen bonds (Figure 3C). Next, we examined the effect of CHE on RRM2 in A549 cells using Western blotting and immunofluorescence. As shown in Figure 3D,E, CHE can decrease RRM2 expression (*p* = 0.0056). To confirm that RRM2 participates in CHE’s inhibition of TGF-β-induced EMT, we conducted further studies using A549 cells transfected with RRM2 over-expression vectors. It was revealed that RRM2 increased cell migration numbers, while CHE could reverse this increase in migration (*p* < 0.01; Figure 3F). These results collectively demonstrate that CHE can directly bind to RRM2 to inhibit TGF-β-induced A549 cell migration.

### 2.4. RRM2 Is Involved in TGF-β-Induced EMT

**Figure 4:**
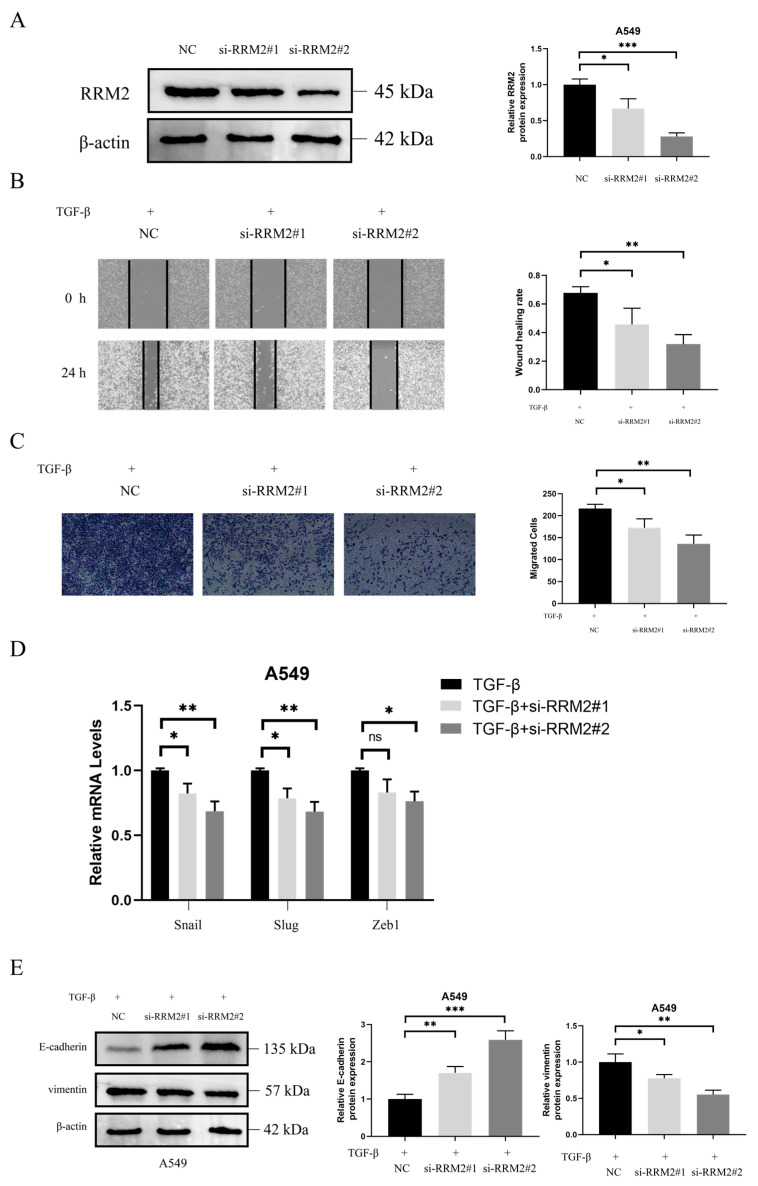
RRM2 participates in TGF-β-induced EMT. (**A**) The transfection efficiency of RRM2 siRNA was measured using Western blot. (**B**,**C**) The effect of RRM2 silencing on the TGF-β-induced migration of A549 cells was evaluated using wound healing and Transwell assays. (**D**) RRM2 silencing affected TGF-β-induced changes in Snail, Slug, and ZEB1 mRNA levels in A549 cells. (**E**) RRM2 silencing affected TGF-β-induced changes in E-cadherin and vimentin expression levels in A549 cells. All scale bars represent 200 µm. Error bar, SD of three independent experiments. For multiple group comparisons, a one-way ANOVA was used. * *p* < 0.05, ** *p* < 0.01, *** *p* < 0.001, ns: non-significance.

In a bioinformatics analysis, we found that RRM2 is highly expressed in lung tumor tissues. Recent studies have indicated that RRM2 plays a significant role in lung cancer [45,46,47]. Our data revealed that CHE can inhibit RRM2 expression in A549 cells. Then, we investigated the role of RRM2 in TGF-β-driven EMT. As demonstrated in Figure 4A, the Western blot results confirmed the successful transfection of RRM2 siRNA in A549 cells (*p* < 0.05). The effects of RRM2 siRNA on TGF-β-driven cell migration were evaluated using wound healing and Transwell assays. As shown in Figure 4B,C, the results indicated that blocking RRM2 significantly inhibited the TGF-β-induced migration of A549 cells (*p* < 0.05). The inhibition of RRM2 also significantly suppressed the TGF-β-induced increase in Slug, Snail, and ZEB1 expression (*p* < 0.05; Figure 4D). As shown in Figure 4E, RRM2 knockdown reversed the TGF-β-induced decrease in E-cadherin expression and the increase in vimentin expression (*p* < 0.05). These results are consistent with the finding that CHE inhibits the TGF-β-induced migration of A549 cells.

### 2.5. CHE Inhibits Cancer Metastasis In Vivo

**Figure 5:**
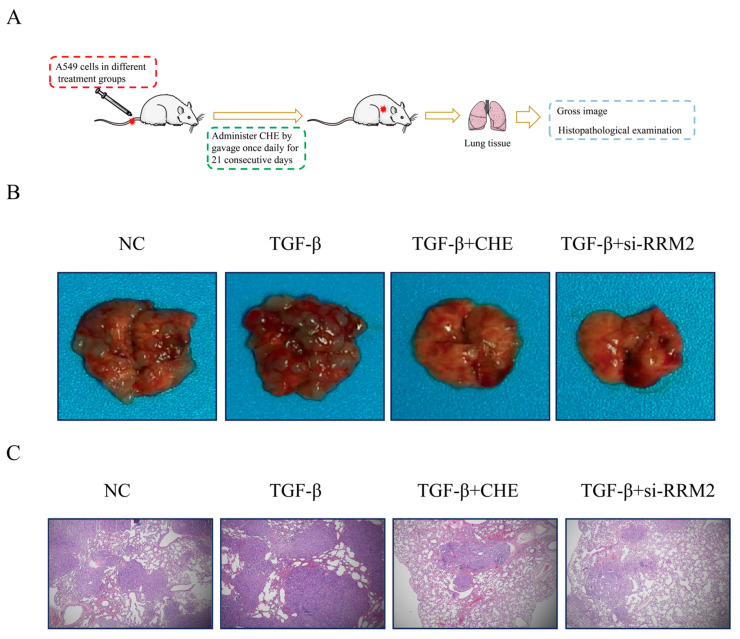
Effects of CHE on TGF-β-enhanced lung metastasis in vivo. (**A**) Schematic diagram of experimental protocol. (**B**) Representative images of lung metastatic nodules. (**C**) Representative images of HE-stained lung tissue (magnification, × 100).

Next, by conducting in vivo experiments, we confirmed that CHE inhibits TGF-β-induced A549 cell transfer by regulating RRM2. A schematic diagram of the animal experiment design is shown in Figure 5A. Representative images of lung metastatic nodules and a lung histological analysis showed that, compared with TGF-β-treated mice, the number, multiplicity, and volume of tumor nodules in the CHE and RRM2 siRNA groups significantly reduced (Figure 5B,C). These data clearly demonstrate that CHE and the loss of RRM2 have anti-metastatic effects on A549 cells pre-treated with TGF-β. Critically, we observed no side effects in mice treated with CHE (Figure S2).

## 3. Discussion

Although recent advancements in cancer therapies have improved initial treatment outcomes, metastasis remains a significant cause of cancer-related mortality. Developing new and more effective natural drugs to treat metastatic disease is essential [48,49]. CHE is a benzophenanthridine-type compound commonly found in plants such as Papaveraceae, Fumariaceae, and Rutaceae. CHE possesses anti-tumor, antibacterial, and anti-inflammatory properties [50]. It has also demonstrated selective cytotoxicity against lung cancer cells [42]. The present research first found that CHE can inhibit TGF-β-induced lung cancer metastasis.

The progression of EMT is regulated by the expression of EMT transcription factors such as Snail, ZEB, and TWIST; miRNAs; and epigenetic and post-translational regulatory factors [51]. An abnormal reactivation of EMT is associated with the malignant properties of tumor cells during cancer progression and metastasis, which includes promoting migration and invasion and enhancing resistance to chemotherapy and immunotherapy [52]. TGF-β, a major driver of EMT, promotes invasiveness and metastasis by inducing EMT [53]. We induced EMT in A549 cells using TGF-β. It was observed that TGF-β increased A549 cell migration; decreased E-cadherin expression; and increased vimentin, Slug, Snail, and ZEB1 expression. Wound healing and Transwell assays indicated that CHE could inhibit the TGF-β-induced migration of A549 cells and reverse changes in EMT biomarkers, including E-cadherin, vimentin, Slug, Snail, and ZEB1. RRM2 is a rate-limiting enzyme involved in DNA synthesis and damage repair, playing a crucial role in various key cellular processes, such as cell proliferation, invasion, migration, and senescence [54]. RRM2 is frequently over-expressed as a tumor driver in various malignancies. Recent studies have indicated that low RRM2 expression can inhibit the development of lung cancer [46,55,56]. However, no studies have investigated the impact of RRM2 on EMT in lung cancer. In our study, we found that silencing RRM2 reduced the migration levels of TGF-β-induced A549 cells, and we observed an increase in E-cadherin expression, along with a decrease in vimentin, Slug, and Snail expression levels. This is consistent with the finding that CHE inhibits TGF-β-induced EMT. Notably, CHE can suppress RRM2 expression. Therefore, we speculate that CHE may inhibit TGF-β-induced EMT by regulating RRM2.

Given the results of the in vitro experiments, we further validated our findings in vivo. We discovered that the CHE and siRRM2 groups could inhibit TGF-β-induced lung cancer metastasis. These results indicate that CHE can inhibit TGF-β-induced lung cancer metastasis and EMT and that this effect is related to the regulation of RRM2.

## 4. Materials and Methods

### 4.1. Reagents and Chemicals

CHE (34316-15-9, purity ≥ 98%) was purchased from Chengdu Dest Biological Technology Co., Ltd. (Sichuan, China). TGF-β was purchased from PeproTech (Rocky Hill, NJ, USA). The primary antibodies for RRM2, E-cadherin, and vimentin were purchased from Cell Signaling Technology (Danvers, MA, USA). β-actin and secondary antibodies were purchased from Protein Technology Group, Inc. (Wuhan, China).

### 4.2. Bioinformatics Analysis

Tumor/normal tissue differential expression and multigene comparative analyses were conducted by searching the Gene Expression Profiling Interactive Analysis (GEPIA) database (http://gepia.cancer-pku.cn/index.html, accessed on 3 June 2022) and the ProteoCancer Analysis Suite (PCAS) database (https://jingle.shinyapps.io/PCAS/, accessed on 4 June 2022).

### 4.3. Molecular Docking

The molecular structure of CHE was sourced from the PubChem database (https://pubchem.ncbi.nlm.nih.gov/, accessed on 20 June 2023). The protein structures of RRM2 were obtained from the AlphaFold Protein Structure database (https://alphafold.ebi.ac.uk/).

AutoDockTools was employed to preprocess the protein receptors and to set the molecular docking parameters. Molecular docking was executed using AutoDock Vina, and the model with the highest binding affinity was selected from the docking results. Visualization of the molecular docking results was achieved using Pymol-2.5.4 software.

### 4.4. Cell Counting Kit-8 (CCK8) Assay

A549 cells were seeded at a density of 5 × 10^3^ cells/well and cultured overnight. After incubation, the cells were treated with different CHE concentrations for 48 h, followed by the addition of 10 μL of CCK8 solution, and absorbance was measured at 450 nm after 2 h.

### 4.5. Cell Culture, siRNA, and Plasmids

A549 cells were acquired from the American Type Culture Collection (Manassas, VA, USA) and cultured at 37 °C in a humidified atmosphere of 5% CO_2_ with RPMI-1640 medium, which was supplemented with 10% fetal bovine serum, 100 U/mL penicillin, and 100 mg/mL streptomycin. The siRNAs of RRM2 were purchased from jtsbio Biotech (jtsbio Biotech Co., Ltd., Wuhan, China) (Supplementary Table S1). MiaoLingPlasmid designed and established the RRM2 over-expression plasmid (transcript variant 1, mRNA NCBI Reference Sequence: NM_001165931.1; MiaoLingPlasmid, Wuhan, China) (Figure S3). Plasmid and siRNA transfection was performed with Lipofectamine^®^ 3000 following the manufacturer’s instructions.

### 4.6. Wound Healing Assay

For a scratch wound healing assay, A549 cells were seeded in 6-well plates at a density of 3 × 10^5^ cells/mL per well. Wounds were created via scratching with a pipette tip. The cells were washed with PBS and then exposed to CHE (2.5 and 5 μM) and TGF-β (10 ng/mL) in culture medium for 24 h. Microscopic images were captured at 0 and 24 h. The migration area in the wound healing assay was quantitatively analyzed by making a comparison with the initial scratch area, and all analyses were performed in three independent experiments.

### 4.7. Transwell Assay

For a Transwell assay, A549 cells were resuspended in serum-free medium at a concentration of 4 × 10^4^ cells/mL and seeded onto the upper surface of the Transwell chamber. The bottom of the chambers was filled with 600 μL of 10% FBS medium containing 2.5 μM or 5 μM CHE. After 4 h of incubation, TGF-β was added to the bottom of the culture medium to a final concentration of 10 ng/mL. After 24 h of incubation, cells remaining on the upper surface of the filter membrane were removed with a cotton swab, while cells that had migrated to the lower surface of the filter membrane were washed with PBS, fixed with 4% paraformaldehyde for 15 min, and then stained with crystal violet solution for 10 min. Images were acquired, and the cells were counted under a Nikon TE-2000U fluorescence microscope.

### 4.8. Quantitative Real-Time PCR

Cells were harvested, and the total RNA was isolated using Trizol reagent (Invitrogen Inc., Carlsbad, CA, USA) according to the manufacturer’s instructions. RNA was quantitated by performing optical density measurements at 260 and 280 nm. Complementary DNA synthesis and qPCR were performed using a TransScript Green Two-Step qPCR SuperMix (TransGen Biotech, Beijing, China). QPCR was performed with a reaction mixture (total volume of 20 µL) that consisted of 2×Trans Start Top Green qPCR SuperMix, Passive Reference Dye, ddH_2_O, cDNA templates, and forward and reverse primers. The amount of Snail, Slug, and ZEB1 mRNA was normalized to GAPDH expression. All of the primers were either ordered from or custom-made by Dingguo Changsheng Biotechnology Co., Ltd, Beijing, China (Table S1). Relative fold changes in the expression of the target gene in the control and other groups were determined using the 2 ^−△△CT^ method.

### 4.9. Immunofluorescence Staining

Cells were washed with PBS, fixed with 4% paraformaldehyde for 15 min, permeabilized with 0.3% Triton-X for 15 min, and blocked with 5% normal goat serum at room temperature for 1 h. Cover slips were incubated with RRM2 antibody overnight at 4 °C. The cells were washed with PBS and incubated with a fluorescence secondary antibody at room temperature for 1 h. After washing with PBS, the cells were stained with DAPI for 5 min and reviewed, and images were acquired with a Nikon TE-2000U fluorescence microscope (Tokyo, Japan).

### 4.10. Histology

Formalin-fixed lung tissue was embedded in paraffin, and 5 μm thick sections were prepared. Sections were mounted on glass slides and stained with hematoxylin and eosin to detect tumors.

### 4.11. Western Blot

Total protein fractions were extracted from cells using RIPA buffer. Proteins were separated via 10% SDS-PAGE and transferred to PVDF membranes, blocked with 5% non-fat milk, washed with TBST, and incubated with primary antibody for 12 h. After washing again, the membranes were incubated with horseradish peroxidase-conjugated secondary antibody for 2 h, followed by another wash. Protein bands were visualized and imaged using ECL reagent, and grayscale values were determined using lmageJ-1.51j8 software.

### 4.12. Mouse Models of Metastasis

Male nude mice aged 6–8 weeks were randomly divided into four groups: a control group (n = 6, where each mouse was injected with A549 cells), a TGF-β group (n = 6, where each mouse was injected with A549 cells treated with TGF-β), a TGF-β + CHE group (n = 6, where each mouse was injected with A549 cells treated with TGF-β and orally administered CHE (30 mg/kg) for 21 days), and a TGF-β + siRRM2 group (n = 6, where each mouse was injected with A549 cells treated with TGF-β after transfection). For cell implantation, A549 cells (1 × 10^6^) were injected via the tail vein. After 21 days, the mice were euthanized, and the lungs were removed and fixed with 10% formalin. Carbon dioxide was used for euthanasia to minimize the animals’ discomfort.

All animal care and experimental procedures were conducted in compliance with the guidelines of the European Community and the Committee for the Care and Use of Laboratory Animals of Jilin Medical University (Jilin, China). The study protocol was approved by the Jilin Medical University Ethics Committee. The study is reported in accordance with ARRIVE guidelines (Animal Ethics Approval Number 2024-GKJJ-019, 11 March 2024).

### 4.13. Analyses

All graphical representations were generated using GraphPad Prism 6. Data are presented as the mean ± standard deviation (SD). Comparisons between two groups were conducted using an unpaired Student’s *t*-test or the Mann–Whitney U test. For multiple group comparisons, a one-way analysis of variance with Tukey’s post hoc test was used. Statistical significance is denoted as follows: * *p* < 0.05, ** *p* < 0.01, and *** *p* < 0.001.

## 5. Conclusions

In summary, we found that CHE inhibits the TGF-β-induced EMT-driven migration and metastasis of A549 cells by regulating RRM2. Advanced metastatic lung cancer exhibits resistance to existing chemotherapy regimens. Fortunately, CHE is a hydrophobic drug that contains many aromatic rings in its molecular structure. As such, it has the potential to self-assemble into nanomedicines, which is certainly advantageous. Our in vitro and in vivo findings indicate that CHE deserves further investigation as a potential candidate for the treatment of lung cancer progression and metastasis.

## Data Availability

The original contributions presented in this study are included in the article/Supplementary Materials. Further inquiries can be directed to the corresponding authors.

## Supplementary Materials

The following supporting information can be downloaded at https://www.mdpi.com/article/10.3390/ph18071036/s1, Figure S1: RRM2 is highly expressed in LUAD; Figure S2: High doses of CHE treatment had no effect on mice liver, spleen, and thymus indices or on serum ALT, AST, BUN, and CREA levels; Figure S3: Construction of RRM2 plasmids; Table S1: Primer sequences used in this study.

## Abbreviations

The following abbreviations are used in this manuscript:

NSCLC	Non-small-cell lung cancer
CHE	Chelerythrine
TGF-β	Transforming growth factor-beta
RRM2	Ribonucleotide reductase subunit M2
EMT	Epithelial–mesenchymal transition
FBS	Fetal bovine serum
DMSO	Dimethyl sulfoxide
GEPIA	Gene expression profiling interactive analysis
PCAS	ProteoCancer analysis suite
DAPI	4′,6-Diamidino-2′-phenylindole
BCA	Bicinchoninic acid
LUAD	Adenocarcinoma
BRCA	Breast invasive carcinoma
CCRCC	Clear cell renal cell carcinoma
OV	Ovarian serous cystadenocarcinoma
