# Supplementary material for: Chelerythrine Inhibits TGF-β-Induced Epithelial–Mesenchymal Transition in A549 Cells via RRM2
Source: Pharmaceuticals (Basel). 2025 Jul 12;18(7):1036. doi: 10.3390/ph18071036 (PMC12298477; doi:10.3390/ph18071036)
Supplement: Supplementary file 1 [file pharmaceuticals-18-01036-s001.zip › pharmaceuticals-3715969-supplementary.pdf]

Supporting Information

Chelerythrine inhibits TGF-β-induced Epithelial–Mesenchymal Transition in A549 cells via RRM2

Jinlong Liu [1,†], Mengran Xu [1,†], Liu Han [1], Yuxuan Rao [1], Haoming Han [1], Haoran Zheng [1], Jinying Wu [1,], and Xin Sun [1,*]*

This file includes:

Figs. S1 to S3

Tables. S1

Supplementary Figures

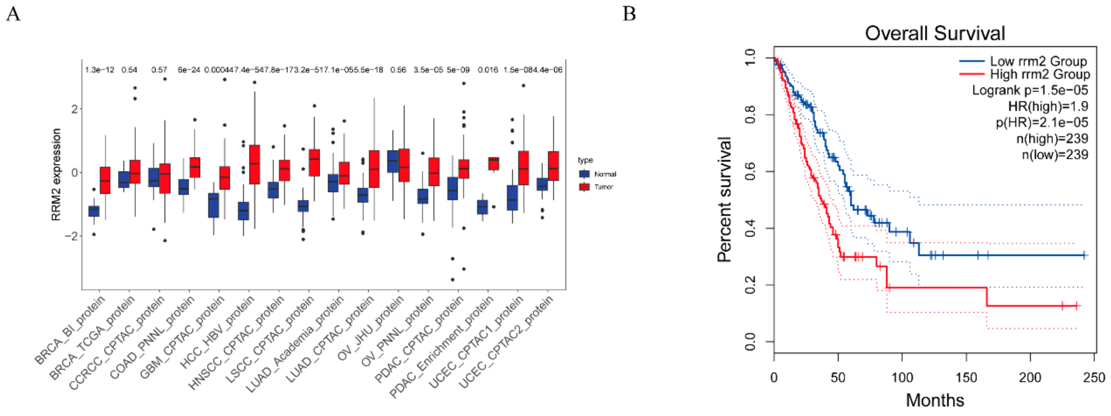

Figure S1. RRM2 is highly expressed in LUAD. (A) Protein level of RRM2 in 16 different kinds of tumors (PCAS). (B) The OS analysis for RRM2 (PCAS).

A

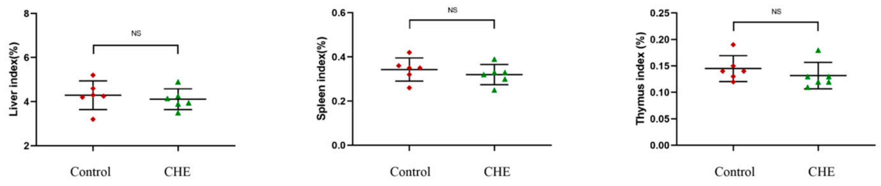

B

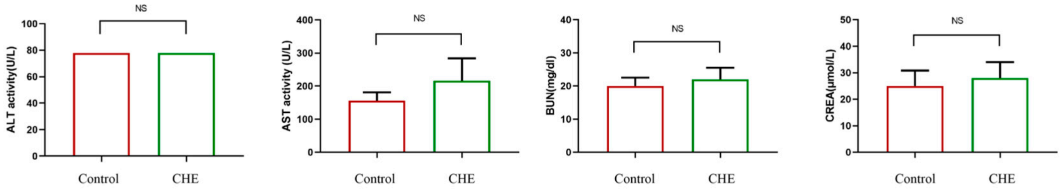

Figure S2. High doses of CHE treatment had no effect on mice liver, spleen, and thymus indices or on serum ALT, AST, BUN, and CREA levels.

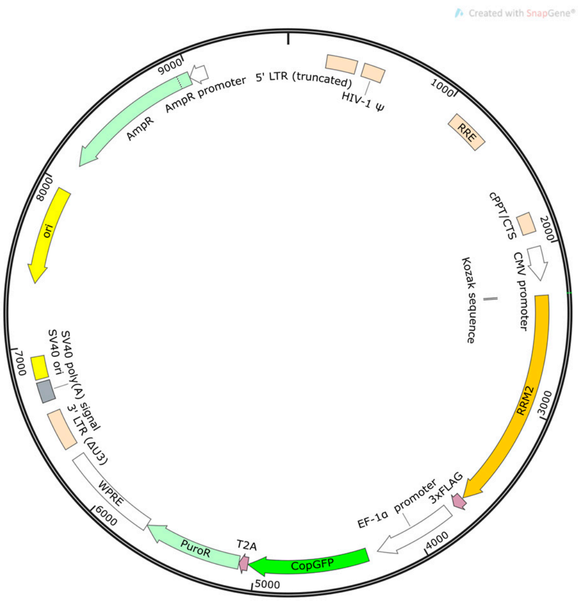

Figure S3. Construction of RRM2 plasmids.

Table S1. Primer sequences used in this study

Reagents	Vendor	Primer sequence（5′-3）
siRNA-RRM2	jtsbio Biotechnology	siRNA-NC<br>Sence: UUCUCCGAACGUGUCACGUTT<br>Anti-Sence: ACGUGACACGUUCGGAGAATT<br>siRNA-RRM2-1<br>Sence: GGCUCAGCUUGGUCGACAATT<br>Anti-Sence: UUGUCGACCAAGCUGAGCCTT<br>siRNA-RRM2-2<br>Sence: GGGAUGAAUUGCACUCUAATT<br>Anti-Sence: UUAGAGUGCAAUUCAUCCCTT
Snail	Beijing Dingguo Changsheng Biotechnology	Forward: TAGCGAGTGGTTCTTCTGCG<br>Reverse: AGATGAGCATTGGCAGCGAG
Slug	Beijing Dingguo Changsheng Biotechnology	Forward: TGGGCTGCCCAAACATAAG<br>Reverse: CCGCAGATCTTGCAAACACA
Zeb1	Beijing Dingguo Changsheng Biotechnology	Forward: AGAGCGCTAGCTGCCAATAA<br>Reverse: GGGCGGTGTAGAATCAGAGT
GAPDH	Beijing Dingguo Changsheng Biotechnology	Forward: AGAAGGCTGGGGCTCATTTG<br>Reverse: AGGGGCCATCCACAGTCTTC
